# Probiotics Alleviate Microcystin-LR-Induced Developmental Toxicity in Zebrafish Larvae

**DOI:** 10.3390/toxics12070527

**Published:** 2024-07-22

**Authors:** Qin Wu, Aoxue Gong, Xixia Liu, Jianjun Hou, Huan Liu, Zhi Yang, Ya Zhu

**Affiliations:** 1 Hubei Key Laboratory of Edible Wild Plants Conservation and Utilization, Huangshi Key Laboratory of Lake Biodiversity and Environmental Conservation, Hubei Normal University, Huangshi 435002, China; 2 Hubei Engineering Research Center of Special Wild Vegetables Breeding and Comprehensive Utilization Technology, Huangshi 435002, China; 3 Key Laboratory of Ministry of Water Resources for Ecological Impacts of Hydraulic Projects and Restoration of Aquatic Ecosystems, Institute of Hydroecology, Ministry of Water Resources & Chinese Academy of Sciences, Wuhan 430079, China; yangzhi4626@163.com; 4 School of Medicine, Taizhou University, Taizhou 318000, China

**Keywords:** Microcystin-LR, probiotics, zebrafish larvae, developmental toxicity

## Abstract

Microcystin-LR (MCLR) poses a significant threat to aquatic ecosystems and public health. This study investigated the protective effects of the probiotic *Lactobacillus rhamnosus* against MCLR-induced developmental toxicity in zebrafish larvae. Zebrafish larvae were exposed to various concentrations of MCLR (0, 0.9, 1.8, and 3.6 mg/L) with or without *L. rhamnosus* from 72 to 168 h post-fertilization (hpf). Probiotic supplementation significantly improved survival, hatching, and growth rates and reduced malformation rates in MCLR-exposed larvae. *L. rhamnosus* alleviated MCLR-induced oxidative stress by reducing reactive oxygen species (ROS) levels and enhancing glutathione (GSH) content and catalase (CAT) activity. Probiotics also mitigated MCLR-induced lipid metabolism disorders by regulating key metabolites (triglycerides, cholesterol, bile acids, and free fatty acids) and gene expression (*ppara*, *pparb*, *srebp1*, and *nr1h4*). Moreover, 16S rRNA sequencing revealed that *L. rhamnosus* modulated the gut microbiome structure and diversity in MCLR-exposed larvae, promoting beneficial genera like *Shewanella* and *Enterobacter* and inhibiting potential pathogens like *Vibrio*. Significant correlations were found between gut microbiota composition and host antioxidant and lipid metabolism parameters. These findings suggest that *L. rhamnosus* exerts protective effects against MCLR toxicity in zebrafish larvae by alleviating oxidative stress, regulating lipid metabolism, and modulating the gut microbiome, providing insights into probiotic-based strategies for mitigating MCLR toxicity in aquatic organisms.

## 1. Introduction

Microcystins (MCs) are produced by cyanobacterial blooms and have become increasingly prevalent in recent years, posing a threat to aquatic environments and human health [1]. MCs are monocyclic heptapeptide compounds, featuring two variable L-amino acids in their chemical structures, allowing for numerous isomeric forms depending on the amino acids present [2]. To date, over 200 MC structures have been identified, among which microcystin-LR (MCLR) is recognized for its potent toxicity and widespread distribution. MCLR is characterized by its physical stability, high-temperature resistance, water solubility, complex structure, and substantial molecular size, contributing to its stable presence in water [3].

The World Health Organization (WHO) recommends safety guidelines for MCs at 1.0 μg/L in drinking water and 10 μg/L in recreational waters [4]. However, natural water bodies frequently exceed these limits, especially during summer cyanobacterial outbreaks. Factors such as increased dissolved carbon dioxide, temperatures, and excessive nutrients in aquatic environments under the backdrop of global change have intensified the persistence, frequency, distribution, and scale of these outbreaks [5]. Studies have detected MCs in aquatic environments worldwide. For instance, an analysis of studies in Latin American countries (2000–2019) revealed MCLR presence in all water types, with an average concentration of up to 0.5 mg/L in multipurpose waters [6]. In surface waters, data from inland Africa from 1989 to 2019 showed relatively high levels of MCLR in the Republic of South Africa, averaging up to 2.8 mg/L [7].

Toxicological studies have reported the significant toxic impact of MCLR on aquatic species, including developmental toxicity [4], hepatotoxicity [8], nephrotoxicity [9], reproductive toxicity [10], neurotoxicity [11,12,13], and immunotoxicity [14]. Aquatic species of economic importance, such as fish, shellfish, and shrimp, are particularly vulnerable to accumulation and poisoning due to prolonged exposure to cyanotoxins in their diet and habitat. MCLR has shown a broad bioaccumulation across trophic levels, highlighting concerns for human food safety.

Given the stability of MCLR in aquatic environments and its enduring negative impact on eutrophic water body ecosystems, the development of in situ ecological remediation strategies to protect aquatic animals from MCLR harm is particularly important and urgent. Probiotics, with their multifaceted bioremediation potential, such as enhancing intestinal barrier integrity, inhibiting inflammation, and promoting detoxification, hold promise as an effective ecological remediation strategy [11,15,16]. However, research on the protective efficacy of probiotics against MCLR toxicity, especially in key aquatic model organisms like zebrafish, remains insufficient.

This study aims to evaluate the potential of probiotics in counteracting the toxicity induced by MCLR. Through acute exposure experiments on zebrafish larvae at different MCLR concentrations (0, 0.9, 1.8, and 3.6 mg/L) and the addition of the probiotic *L. rhamnosus*, this study comprehensively assessed growth indicators at 168 h post-fertilization (hpf), including weight, length, weight gain, and specific growth rate. Moreover, the study explored the interactions between MCLR and probiotics on oxidative stress and antioxidant activity and analyzed changes in lipid metabolism, covering metabolite contents, fluorescence imaging, and transcriptional changes in nuclear receptor genes. By analyzing the intestinal microbiome composition, the study further established correlations with host biochemical parameters. Accordingly, this study aims to reveal the potential efficacy of probiotics in alleviating developmental defects in zebrafish caused by MCLR, providing a scientific basis for developing in situ ecological remediation strategies.

## 2. Materials and Methods

### 2.1. Exposure Experiments

Adult male and female zebrafish (*Danio rerio*) were acclimated and maintained in a controlled environment at 28 ± 0.5 °C with a 14-h light/10-h dark cycle, as previously described [17,18]. The day before mating, male and female zebrafish were separated in spawning boxes. The following morning, the divider was removed and spawning was induced by light stimulation. Fertilized eggs were collected and cultured under standard conditions until 72 hpf for exposure experiments.

MCLR (purity >95%, Express Technology Co. Ltd., Taipei, China) was dissolved in ultrapure water to prepare a 500 mg/L stock solution and stored at low temperatures in the dark. The probiotic *Lactobacillus rhamnosus* GG (GDMCC 1.2223) was kindly provided by the Guangdong Institute of Microbiology (Guangzhou, China). All other chemicals were of analytical grade.

At 72 hpf, the culture solution was replaced with exposure solutions of different concentrations. Zebrafish larvae were randomly divided into eight groups: blank control, probiotic control (1 × 10^6^ CFU/mL), three MCLR exposure groups (0.9, 1.8, and 3.6 mg/L), and their corresponding MCLR+probiotic groups. Each group had three replicates with approximately 300 larvae per replicate, placed in beakers containing 200 mL of exposure medium. The exposure medium was renewed daily to maintain stable MCLR concentrations and water quality. During the exposure period, larvae were not fed to eliminate the potential influence of food on the results. The exposure experiment lasted until 168 hpf, after which samples were collected and stored at −80 °C for further analysis.

### 2.2. Determination of MCLR Concentrations

The method used for determining the concentration of MCLR in water samples was based on previous research [1]. An enzyme-linked immunosorbent assay (ELISA) kit (Beacon Analytical Systems Inc., Saco, ME, USA, product number: 20-0068) was employed following the manufacturer’s instructions. The recovery rate and the relative standard deviation (RSD) of this method were 110.04% and 4.7%.

The measured concentrations of MCLR in water samples with nominal concentrations of 0, 0.9, 1.8, and 3.6 mg/L were ND, 0.87 ± 0.05, 1.92 ± 0.08, and 3.41 ± 0.12 mg/L, respectively.

### 2.3. Growth and Development of Zebrafish Larvae

During the single or combined exposure period, larval mortality, hatching rates, and deformities (such as pericardial edema, yolk sac edema, and body curvature) were meticulously monitored and recorded on a daily basis. To accurately measure body weight at 168 hpf, larvae were initially placed on Kimwipes to remove excess water, following which ten larvae from the same culture dish were transferred to a test tube. This procedure, considering the zebrafish larvae’s small body volume, facilitates more precise weight measurements using a precision balance (Mettler Toledo, Columbus, OH, USA) with three replicates per experimental group. Dorsal images of the larvae were obtained with a stereomicroscope equipped with a camera to measure body length, involving fifteen larvae per group. Body weight increase was calculated as (W2–W1)/W1 %, based on the initial body weight (W1) at 72 hpf and the final body weight (W2) at 168 hpf. Additionally, the specific growth rate was determined using the formula (ln W2–ln W1)/T %, where T denotes the observation period.

### 2.4. Evaluation of Oxidative Stress and Lipid Metabolism Markers

Upon completion of the exposure experiment, approximately 50 larvae at 168 hpf were randomly sampled from each beaker and pooled to form one biological replicate (*n* = 6 replicates per exposure group). The larval samples were homogenized in ice-cold physiological saline, followed by centrifugation at 6000× *g* for 10 min at 4 °C. The resulting supernatants were collected for subsequent analyses of oxidative stress and lipid metabolism markers.

Reactive oxygen species (ROS) content was quantified using the 2′,7′-dichlorofluorescin diacetate probe through fluorescence detection. The concentration of glutathione (GSH), enzymatic activities of superoxide dismutase (SOD) and catalase (CAT), and concentrations of key metabolites involved in lipid metabolism, including triglycerides (TG), free fatty acids (FFA), glycerol, bile acids, cholesterol, and lipid peroxidation, were determined. All these biochemical evaluations were examined by using commercially available colorimetric assay kits (Nanjing Jiancheng Bioengineering Institute, Nanjing, China) in accordance with the manufacturer’s instructions.

### 2.5. Lipid Visualization via BODIPY 505/515 Fluorescence

Lipid quantification and spatial distribution in zebrafish larvae were investigated using a fluorescence-based staining approach. Following acute exposure, fifteen larvae from each experimental group at 168 hpf were randomly selected and stained with 100 μM BODIPY 505/515 dye (Thermo Fisher Scientific, Waltham, MA, USA). The staining procedure was conducted in the dark for one hour. Post-staining, larvae were anesthetized and examined under a Nikon fluorescence microscope (Nikon Inc., Melville, NY, USA) with excitation and emission wavelengths set to 505 nm and 515 nm, respectively. Quantification of fluorescence intensity across the entire larva was performed using ImageJ software (version 1.53k, National Institutes of Health, Bethesda, MD, USA). To avoid potential interference from non-specific signals in tissues such as eyes and lenses, the regions of interest (ROIs) for fluorescence quantification were specifically defined to include only the intestine and gallbladder. During image acquisition, zebrafish larvae were consistently positioned and the same exposure intensity was used for all images to minimize measurement errors.

### 2.6. Quantitative Real-Time Polymerase Chain Reaction (qRT-PCR) Analysis

The details of primer sequences for the genes under study are presented in Table S1 of the Supporting Information. For normalization purposes, *gapdh* (glyceraldehyde 3-phosphate dehydrogenase) was utilized as the reference gene [19]. Total RNA extraction, first-strand cDNA synthesis, and qRT-PCR assays were performed according to standard protocols. Total RNA was extracted from zebrafish samples using TRIzol (Takara, Kusatsu, Japan) and first-strand cDNA was synthesized using the PrimeScript^TM^ RT Master Mix (Takara, Kusatsu, Japan) following the manufacturer’s instructions. The qRT-PCR experiments were carried out using the Applied Biosystems StepOnePlus Real-Time PCR System (Foster City, CA, USA). The PCR conditions were programmed to include an initial denaturation at 95 °C for 30 s, followed by 40 amplification cycles of 95 °C for 5 s and 60 °C for 30 s. Relative gene expression was quantified using the 2^−ΔΔCT^ method [20,21].

### 2.7. Bacterial 16S rRNA Gene Sequencing

The gut microbiome compositions of zebrafish larvae exposed to MCLR and/or probiotics were analyzed using 16S rRNA gene sequencing. Approximately 100 larvae were randomly selected from each experimental group and combined into a single sample for analysis (with three such combined samples per treatment group, denoted as *n* = 3). Total genomic DNA was extracted using the DNeasy Blood and Tissue Kit (Qiagen, Hilden, Germany). This DNA served as a template in PCRs, employing the primers 341F (5′-CCTAYGGGRBGCASCAG-3′) and 806R (5′-GGACTACNNGGGTATCTAAT-3′) to target and amplify the V3-V4 regions of the bacterial 16S rRNA gene specifically. Subsequent sequencing of these PCR products was carried out on the Illumina MiSeq platform (Illumina, San Diego, CA, USA), generating 250 base-pair reads in a paired-end format. Following a rigorous quality control process and the removal of sequences not meeting the quality criteria, Operational Taxonomic Units (OTUs) were aggregated based on a sequence similarity threshold of 97%, utilizing the UPARSE 7.1 algorithm. Each OTU was then assigned a representative sequence for taxonomic classification through the RDP classifier (version 2.2), referencing the Green Genes Database. Additionally, sequence counts were aggregated across various taxonomic strata, notably at the phylum and genus levels. Hierarchical clustering and principal co-ordinate analysis (PCoA) were then applied to assess the distribution patterns of genera, showing a relative abundance of over 1% in any of the samples. Spearman’s correlation analysis was used to identify significant associations (coefficients over 0.6 or less than −0.6, *p*-value less than 0.01) between microbiome taxa and biochemical markers.

### 2.8. Data Analysis

Results were presented as mean ± standard error of the mean (SEM). Statistical analyses were performed using SPSS Statistics software (version 22.0, IBM Corporation, Armonk, NY, USA). Prior to analysis, data were examined for normality using the Shapiro–Wilk test and homogeneity of variances using Levene’s test. All data sets met these assumptions. One-way analysis of variance (ANOVA) followed by Tukey’s post-hoc test was used to determine statistical significance. Student’s *t*-test was simultaneously employed to assess the differences between MCLR exposure groups and MCLR+ probiotic groups, which was set at *p* < 0.05.

## 3. Results and Discussion

### 3.1. Probiotics Rescue Zebrafish Larvae from MCLR-Induced Growth Retardation

**Figure 1:**
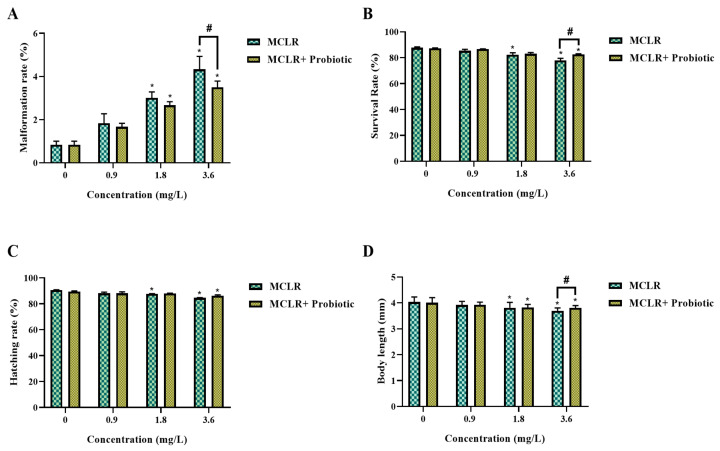
Changes in growth parameters of zebrafish larvae exposed to 0, 0.9, 1.8, and 3.6 mg/L MCLR with or without probiotic supplementation. (**A**) Malformation rate; (**B**) Survival rate; (**C**) Hatching rate; (**D**) Body length. Data are presented as the mean ± SEM of replicates. * *p* < 0.05 indicates significant differences between the exposed groups and the control group; ^#^ *p* < 0.05 indicates significant differences between the co-exposed groups and the corresponding MCLR-only exposed groups.

**Figure 2:**
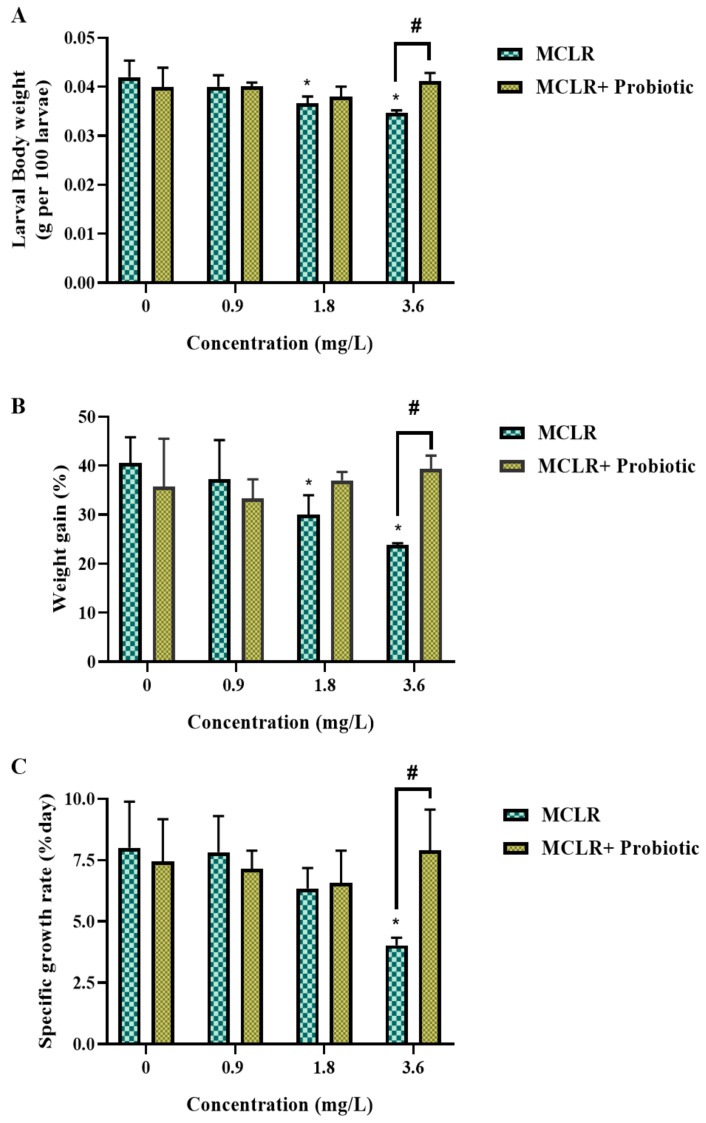
Changes in growth parameters of zebrafish larvae exposed to 0, 0.9, 1.8, and 3.6 mg/L MCLR with or without probiotic supplementation. (**A**) Body weight; (**B**) Weight gain; (**C**) Specific growth rate. Data are presented as the mean ± SEM of replicates. * *p* < 0.05 indicates significant differences between the exposed groups and the control group; ^#^ *p* < 0.05 indicates significant differences between the co-exposed groups and the corresponding MCLR-only exposed groups.

In the present study, we evaluated the impact of MCLR exposure on zebrafish larval development and investigated whether probiotic supplementation could mitigate MCLR toxicity. Zebrafish larvae were exposed to 0, 0.9, 1.8, and 3.6 mg/L MCLR and the results revealed a dose-dependent effect of MCLR on larval growth and survival. The malformation rate increased with elevated MCLR concentrations, reaching the highest level at 3.6 mg/L. In contrast, probiotic supplementation significantly reduced the malformation rate in the 3.6 mg/L MCLR treatment group (Figure 1A). Furthermore, the survival rate of larvae decreased with increasing MCLR concentrations in the MCLR-only treatment groups. However, a significant improvement in survival rate was observed in the probiotic co-exposure groups, particularly at high concentrations of 1.8 and 3.6 mg/L MCLR (Figure 1B). The hatching rate also declined with increasing MCLR exposure concentrations but probiotic supplementation restored the hatching rate to a normal level at 1.8 mg/L MCLR (Figure 1C). Both body length and weight of larvae were reduced under MCLR exposure, while this adverse effect was significantly alleviated by probiotic protection, with recovery observed in terms of the body length and weight at 3.6 mg/L MCLR (Figure 1D and Figure 2A). Moreover, the percentage of body weight gain in larvae was significantly decreased under 1.8 and 3.6 mg/L MCLR exposure, while in the probiotic co-exposure groups, body weight gain was restored to control levels at all concentrations (Figure 2B). The specific growth rate showed a similar trend, with a significant reduction at 3.6 mg/L MCLR treatment and recovery in the probiotic co-exposure group (Figure 2C). These data suggest that probiotic supplementation can significantly attenuate the growth inhibition effects of MCLR on zebrafish larvae, potentially by improving survival, reducing malformation occurrence, and enhancing growth performance.

### 3.2. Probiotics Modulate MCLR-Induced Oxidative Stress and Enhance the Antioxidant Response in Zebrafish Larvae

**Figure 3:**
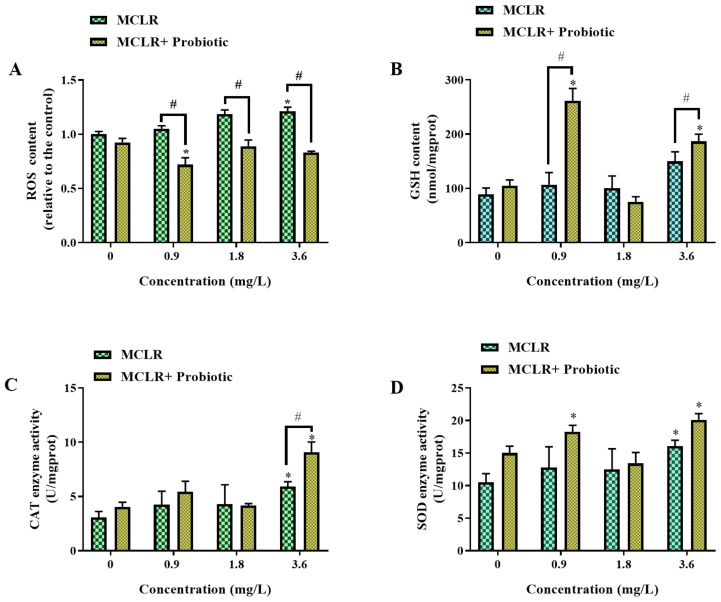
Changes in oxidative stress biomarkers of zebrafish larvae exposed to 0, 0.9, 1.8, and 3.6 mg/L MCLR with or without probiotic supplementation. (**A**) Reactive oxygen species (ROS) content, (**B**) Glutathione (GSH) content, (**C**) Catalase (CAT) activity, and (**D**) Superoxide dismutase (SOD) activity. Data are presented as the mean ± SEM of replicates. * *p* < 0.05 indicates significant differences between the exposed groups and the control group; ^#^ *p* < 0.05 indicates significant differences between the co-exposed groups and the corresponding MCLR-only exposed groups.

This study investigated the oxidative stress response in zebrafish larvae under MCLR exposure and evaluated the modulatory effects of probiotic supplementation at different MCLR concentrations (0, 0.9, 1.8, and 3.6 mg/L). The biomarkers measured included the ROS content, GSH level, CAT activity, and SOD activity (Figure 3).

ROS content showed statistical significance only in the 3.6 mg/L MCLR group. However, probiotic supplementation significantly reduced the ROS levels at 0.9, 1.8, and 3.6 mg/L MCLR, indicating that probiotics can effectively alleviate MCLR-induced oxidative stress (Figure 3A). Probiotics are live microorganisms with potential health benefits and their mechanisms include enhancing host antioxidant defense and reducing oxidative stress levels [22]. Furthermore, probiotic supplementation significantly increased the GSH content in zebrafish larvae at 0.9 and 3.6 mg/L MCLR exposure compared to their respective MCLR-only treatment groups (Figure 3B), suggesting that probiotics can enhance the antioxidant defense capacity of the organism. GSH is a crucial intracellular antioxidant that scavenges free radicals and maintains cellular redox balance [23]. Regarding antioxidant enzyme activities, probiotic supplementation significantly elevated CAT activity at 3.6 mg/L MCLR exposure (Figure 3C). CAT is a key antioxidant enzyme that converts hydrogen peroxide into water and oxygen, protecting cells from oxidative damage [24]. Therefore, the ability of probiotics to increase GSH levels and CAT activity may be an important mechanism for their attenuation of MCLR toxicity. Notably, SOD activity was significantly increased in the probiotic + 0.9 mg/L MCLR co-treatment group, 3.6 mg/L MCLR-only exposure group, and probiotic + 3.6 mg/L MCLR co-treatment group compared to the control, indicating that high concentration MCLR exposure can induce SOD activity and that probiotics may have a synergistic effect. SOD is an important antioxidant enzyme that converts the superoxide anion to hydrogen peroxide and oxygen [25]. The increase in SOD activity may reflect an adaptive response of the organism to MCLR-induced oxidative stress. In summary, the results of this study demonstrate that probiotic supplementation can alleviate MCLR-induced oxidative injury.

### 3.3. Probiotics Mitigate MCLR-Induced Alterations in the Lipid Metabolism of Zebrafish Larvae

**Figure 4:**
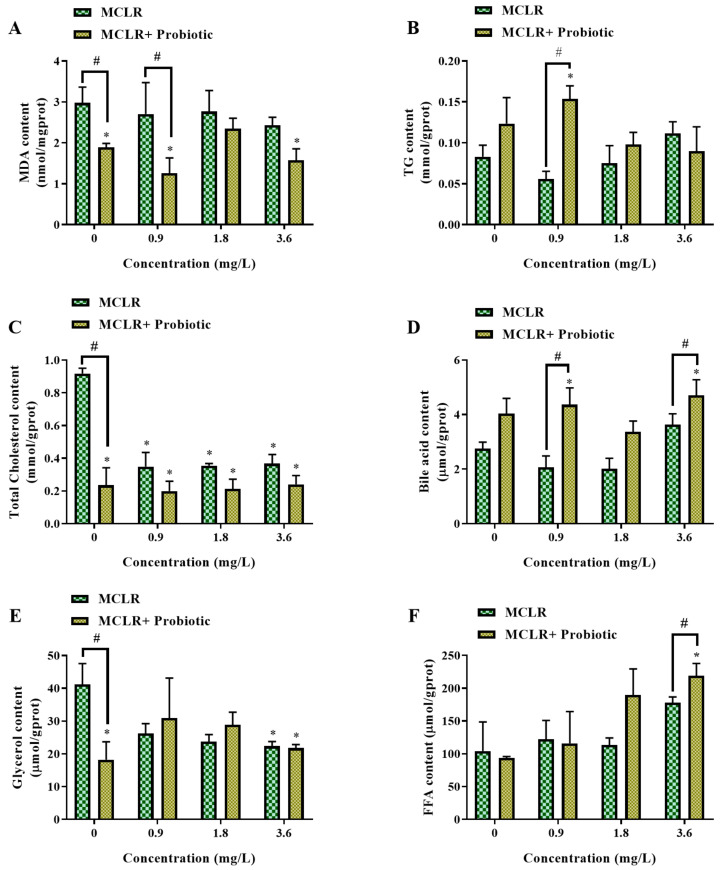
Changes in lipid-related biomarkers of zebrafish larvae exposed to 0, 0.9, 1.8, and 3.6 mg/L MCLR with or without probiotic supplementation. (**A**) Malondialdehyde (MDA) content, (**B**) Triglyceride (TG) content, (**C**) Total cholesterol content, (**D**) Bile acid content, (**E**) Glycerol content, and (**F**) Free fatty acid (FFA) content. Data are presented as the mean ± SEM of replicates. * *p* < 0.05 indicates significant differences between the exposed groups and the control group; ^#^ *p* < 0.05 indicates significant differences between the co-exposed groups and the corresponding MCLR-only exposed groups.

In this study, we evaluated the effects of MCLR and/or probiotic exposure on lipid metabolism in zebrafish larvae and measured a series of lipid metabolites, including TG, FFA, glycerol, total cholesterol, total bile acids, and malondialdehyde (MDA) (Figure 4).

Compared to the control group, probiotic treatment alone significantly reduced MDA content in larvae (Figure 4A), indicating that probiotics have an anti-lipid peroxidation effect, which may help protect larvae from oxidative damage and promote normal growth and development. This is consistent with previous reports on the role of probiotics in reducing lipid peroxidation levels [26,27]. In the MCLR-only exposure groups, no significant changes were observed in the levels of various lipid metabolites except for a significant decrease in the total cholesterol content at all concentrations compared to the control group. However, in the 0.9 mg/L MCLR exposure group, probiotic supplementation significantly increased the TG content (Figure 4B), which may be related to the promotion of fatty acid synthesis and TG accumulation by probiotics [28], providing a basis for energy storage and growth in larvae. In addition, probiotic supplementation significantly reduced total cholesterol levels in most MCLR exposure groups (Figure 4C), while significantly increasing total bile acid content at all MCLR exposure concentrations (Figure 4D). This suggests that probiotics may regulate cholesterol homeostasis by promoting the conversion of cholesterol to bile acids [16] and that the increase in bile acids may help promote lipid digestion and absorption, providing essential nutrients for larval growth and development. Notably, in the 3.6 mg/L MCLR exposure group, the addition of probiotics significantly increased FFA levels (Figure 4F). Considering the previously observed changes in TG content, we speculate that the elevation in FFA levels may reflect the comprehensive regulatory effects of probiotics on lipid metabolism. Under high concentration MCLR stress, probiotics may promote fatty acid synthesis to maintain TG levels, providing energy reserves for larvae. On the other hand, probiotics may also enhance fatty acid mobilization and utilization to meet energy demands, leading to increased FFA levels. This coordinated regulation of lipid synthesis and breakdown metabolism may be a key mechanism by which probiotics maintain energy homeostasis and promote normal growth and development in larvae.

**Figure 5:**
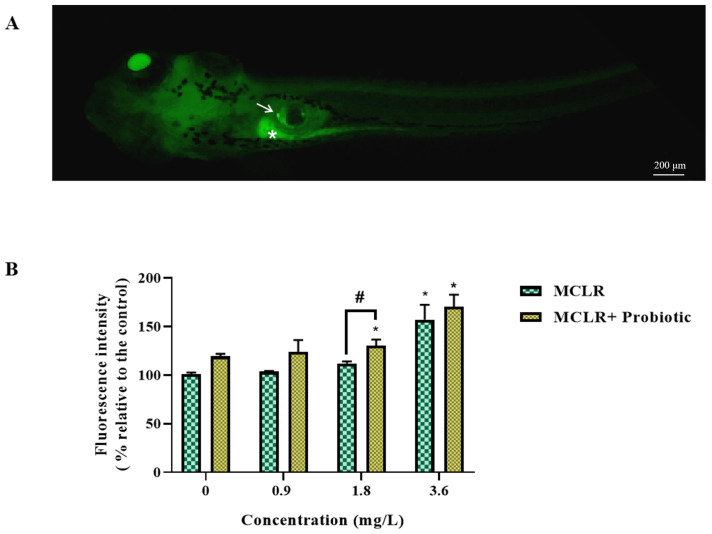
Representative graph of lipid staining (**A**) and quantitative intensity of fluorescence signal (**B**) in zebrafish larvae exposed to 0, 0.9, 1.8, and 3.6 mg/L MCLR with or without probiotic supplementation. Values are presented as mean ± SEM of replicates. * *p* < 0.05 indicates significant differences between the exposed groups and the control group; ^#^ *p* < 0.05 indicates significant differences between the co-exposed groups and the corresponding MCLR-only exposed groups. The arrow points to the gallbladder and the asterisk points to the intestine.

To further elucidate the effects of MCLR and probiotics on lipid accumulation, we performed a visualization analysis of neutral lipids in zebrafish larvae using BODIPY fluorescent dye. As shown in Figure 5A, lipids were mainly distributed in the gallbladder and intestine, consistent with previous studies [15], indicating that these sites are the main locations for lipid digestion, absorption, and storage [29], which are crucial for larval growth and development. Quantitative analysis showed that, in the 1.8 and 3.6 mg/L MCLR exposure groups, the addition of probiotics significantly enhanced lipid fluorescence signals (Figure 5B). This suggests that probiotics may alleviate MCLR-induced lipid metabolism disorders by promoting lipid absorption and transport, thereby providing a material and energy basis for larval growth and development.

**Figure 6:**
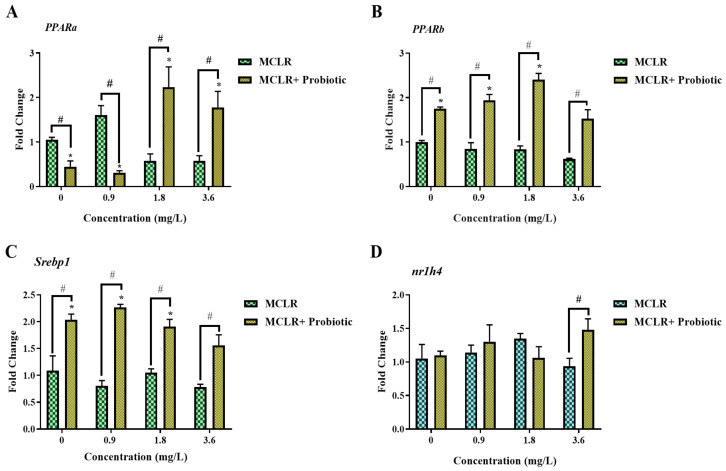
Changes in the expression of genes related to lipid metabolism pathways in zebrafish larvae exposed to 0, 0.9, 1.8, and 3.6 mg/L MCLR with or without probiotic supplementation. (**A**) Peroxisome proliferator-activated receptor alpha (*PPARa*), (**B**) Peroxisome proliferator-activated receptor beta (*PPARb*), (**C**) Sterol regulatory element-binding protein 1 (*srebp1*), and (**D**) Nuclear receptor subfamily 1, group H, member 4 (*nr1h4*). Data are presented as the mean ± SEM of replicates. * *p* < 0.05 indicates significant differences between the exposed groups and the control group; ^#^ *p* < 0.05 indicates significant differences between the co-exposed groups and the corresponding MCLR-only exposed groups.

To clarify the molecular mechanisms by which MCLR and probiotics regulate lipid metabolism, we measured the gene expression levels of lipid metabolism-related nuclear receptors. Peroxisome proliferator-activated receptors α (*ppara*) and β (*pparb*) are key transcription factors involved in fatty acid β-oxidation and energy homeostasis regulation [30], playing important roles in growth and development. This study found that probiotic supplementation down-regulated *ppara* mRNA levels in the 0 and 0.9 mg/L MCLR exposure groups while up-regulating *ppara* expression in the 1.8 and 3.6 mg/L MCLR exposure groups (Figure 6A). Considering the important role of *ppara* in fatty acid oxidation metabolism, this differential regulation may reflect the adaptive regulation of lipid metabolism by probiotics under different MCLR stress intensities.

Combined with the lipid metabolite analysis results, we found that in the 0.9 mg/L MCLR exposure group, probiotic supplementation significantly increased the TG content (Figure 4B), indicating that probiotic supplementation enhanced lipid synthesis metabolism. In this case, down-regulation of *ppara* expression may help reduce fatty acid oxidation and promote TG accumulation, providing energy reserves for larval growth and development. In contrast, in the 3.6 mg/L MCLR exposure group, the addition of probiotics significantly increased the FFA levels (Figure 4F), suggesting increased fatty acid mobilization and utilization. Under this high concentration MCLR stress, up-regulation of *ppara* expression may help promote FFA oxidative metabolism, providing more energy to cope with the stress. In contrast, probiotics significantly up-regulated *pparb* mRNA levels in the 0.9, 1.8, and 3.6 mg/L MCLR exposure groups (Figure 6B). Considering the important role of *pparb* in fatty acid synthesis metabolism [31], its up-regulated expression may reflect the regulatory effect of probiotics on lipid synthesis. This is consistent with our previous hypothesis that probiotics promote fatty acid synthesis under high-concentration MCLR stress. Similarly, the change in the expression trend of sterol regulatory element-binding protein 1 (*srebp1*) was consistent with that of *pparb* (Figure 6C), suggesting that probiotics may promote lipid synthesis metabolism by activating the Srebp1 signaling pathway [32], providing raw materials for cell membrane synthesis and growth and development. In addition, in the 3.6 mg/L MCLR exposure group, probiotics significantly upregulated the mRNA level of the bile acid receptor farnesoid X receptor (*nr1h4*) (Figure 6D). *nr1h4* is a key nuclear receptor that regulates bile acid synthesis and transport [33] and its up-regulated expression may be related to the promotion of bile acid synthesis and excretion by probiotics, improving lipid digestion and absorption and thus promoting growth and development.

In summary, probiotic supplementation can alleviate MCLR-induced lipid metabolism disorders by regulating the levels of key lipid metabolites (TG, total cholesterol, total bile acids, and FFA) and the expression of lipid metabolism-related genes (*ppara*, *pparb*, *srebp1*, and *nr1h4*), thereby improving larval growth and development.

### 3.4. Probiotics and MCLR Differentially Sculpt the Gut Microbiome Structure in Zebrafish Larvae

This study employed 16S rRNA sequencing technology to analyze the effects of MCLR exposure and probiotic intervention on the composition and diversity of the gut microbiota in zebrafish larvae and explored the correlations between gut microbiota and host physiological parameters to elucidate the potential mechanisms by which probiotics alleviate MCLR toxicity.

**Figure 7:**
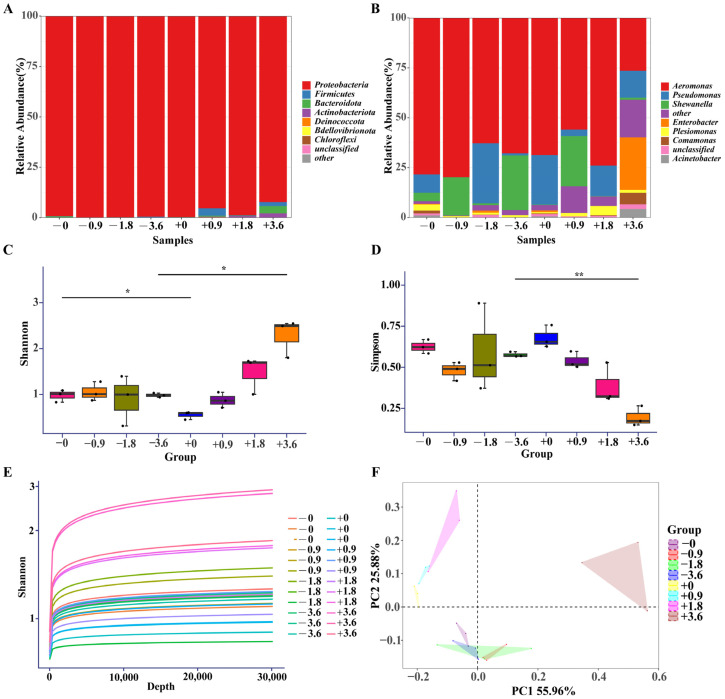
Composition and diversity analysis of the gut microbiome in zebrafish larvae exposed to 0, 0.9, 1.8, and 3.6 mg/L MCLR with (+) or without (−) probiotic supplementation. Rarefaction curves of observed OTUs (**A**). Relative abundance of the gut microbiome at the phylum level (**B**) and genus level (**C**). Alpha diversity indices include the Shannon index (**D**) and Simpson index (**E**). Principal Coordinate Analysis (PCoA) of beta diversity based on the Bray–Curtis distance (**F**). * *p* < 0.05, ** *p* < 0.01 indicates significant differences between the treatment groups.

To assess the quality of the 16S rRNA sequencing data in this study, we plotted rarefaction curves (Figure 7E). The results showed that as sequencing depth increased, the number of OTUs gradually plateaued and reached saturation, indicating sufficient sequencing depth and coverage, high data reliability, and suitability for subsequent bioinformatic analyses [34,35]. At the phylum level, *Proteobacteria* was the dominant bacterial group in all samples (Figure 7A), consistent with previous studies on the composition of the zebrafish gut microbiota [36]. However, co-treatment with high concentrations of MCLR (1.8 and 3.6 mg/L) and probiotics significantly increased the relative abundance of *Firmicutes* and *Bacteroidota*. *Firmicutes* and *Bacteroidota* are important taxa in the fish gut microbiota, playing crucial roles in host nutrient metabolism, immune regulation, and resistance to pathogen colonization [37]. Therefore, the promotion of *Firmicutes* and *Bacteroidota* growth by probiotics in the gut of zebrafish larvae under high-concentration MCLR stress may be one of the important mechanisms by which probiotics alleviate MCLR toxicity.

At the genus level, *Aeromonas* was the dominant genus in each treatment group (Figure 7B). Notably, probiotic supplementation significantly increased the relative abundance of *Shewanella* and *Enterobacter* under MCLR stress. *Shewanella* is a genus with strong heavy metal reduction ability and has broad application prospects in environmental bioremediation [38]. *Enterobacter* has been reported to have the characteristics of producing antibiotics and degrading organic pollutants [39,40]. Therefore, the promotion of the growth of these two genera by probiotics under MCLR stress may help alleviate the toxic effects of MCLR. In addition, the abundance of *Pseudomonas* decreased with increasing concentration in the MCLR-only treatment groups, while in the MCLR and probiotic co-treatment groups, although the overall abundance was low, it increased with increasing MCLR concentration, suggesting that probiotics may affect the host response to MCLR by regulating the growth of *Pseudomonas*.

Alpha diversity analysis revealed that MCLR exposure did not significantly alter the gut microbiota diversity in zebrafish larvae compared to the control group. However, under high concentration MCLR stress (3.6 mg/L), probiotic supplementation significantly enhanced the gut microbiota diversity, as evidenced by a higher Shannon index (Figure 7C) and a lower Simpson index (Figure 7D) compared to the MCLR-only treatment group. Gut microbiota diversity is considered an important factor and indicator influencing host health [41]. A highly diverse gut microbiota can provide more comprehensive metabolic functions and enhance the adaptability of the host to environmental stress [42]. Therefore, the improvement in gut microbiota diversity by probiotics under high concentration MCLR stress may be one of the important mechanisms for their gut protective effects. Beta diversity PCoA analysis revealed that the gut microbiota structure of the MCLR-only treatment group samples was similar, while the addition of probiotics significantly changed the compositional characteristics of the gut microbiota in zebrafish larvae under MCLR stress (Figure 7F), further indicating that the reconstruction of the gut microbiota by probiotics may be a key link in their intervention in MCLR toxicity.

**Figure 8:**
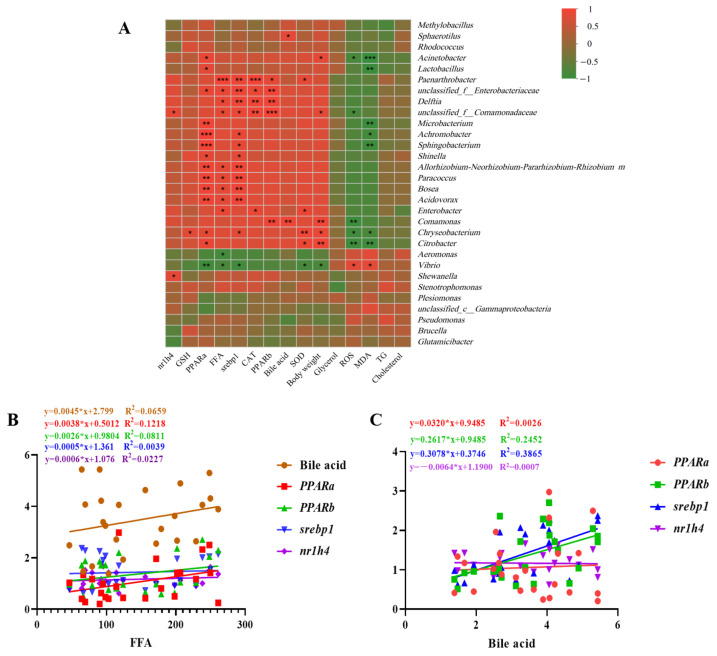
Correlation analysis of the gut microbiome, host physiological parameters, and gene expression levels in zebrafish larvae exposed to MCLR with or without probiotic supplementation. (**A**) Heatmap of Spearman’s correlation coefficients between dominant bacterial genera and biochemical markers, as well as gene expression levels. Red represents a positive correlation, while green represents a negative correlation. (**B**) Correlation analysis between FFA content and bile acid content, as well as the expression of genes related to lipid metabolism (*PPARa*, *PPARb*, *srebp1*, and *nr1h4*). (**C**) Correlation analysis between bile acid content and the expression of *PPARa*, *PPARb*, *srebp1*, and *nr1h4*. * *p* < 0.05, ** *p* < 0.01, *** *p* < 0.001.

To further understand the biological significance of probiotic regulation of the gut microbiota, this study analyzed the correlations between bacterial taxa and host physiological parameters (Figure 8). The results showed that the abundance of several bacterial genera, including *Lactobacillus*, unclassified *Comamonadaceae*, and *Chryseobacterium*, was positively correlated with multiple host parameters related to antioxidant capacity (GSH, CAT, and SOD), lipid metabolism (FFA, *ppara*, and *srebp1*), and growth (body weight) (Figure 8A). These findings suggest that these bacterial taxa may synergistically enhance host functions through various pathways, potentially contributing to the alleviation of MCLR toxicity by probiotics.

Interestingly, the unclassified abundances of *Comamonadaceae* and *Shewanella* were positively correlated with *nr1h4* gene expression (Figure 8A). Moreover, the abundance of *Sphaerotilus* and *Comamonas* was positively correlated with bile acid levels, which were further positively associated with the expression of *pparb* and *srebp1* (Figure 8C). These findings suggest that these bacterial genera may indirectly influence host lipid metabolism through their association with bile acids and the modulation of nuclear receptor expression, potentially contributing to the amelioration of MCLR-induced metabolic disorders by probiotics. In contrast, the abundance of *Vibrio*, which is known to include some opportunistic pathogenic species [43], was positively correlated with oxidative stress (ROS) and lipid peroxidation (MDA) indicators and negatively correlated with antioxidant capacity (SOD), growth (body weight), and lipid metabolism (FFA) parameters (Figure 8A). These results indicate that certain species within these genera may exacerbate MCLR toxicity by inducing oxidative damage, disrupting lipid metabolism and inhibiting growth. Notably, probiotics were found to significantly inhibit these potentially harmful bacteria, thereby exerting a gut protective effect.

## 4. Conclusions

This study demonstrates that the probiotic *L. rhamnosus* can effectively alleviate MCLR-induced developmental toxicity in zebrafish larvae. Probiotic supplementation significantly improved larval survival, hatching, and growth performance and reduced malformation rates under MCLR stress. *L. rhamnosus* attenuated MCLR-induced oxidative damage by reducing ROS levels and enhancing antioxidant defense capacity. Furthermore, probiotics mitigated MCLR-induced lipid metabolism disorders by modulating key lipid metabolites (triglycerides, cholesterol, bile acids, and free fatty acids) and regulating the expression of lipid metabolism-related genes (*ppara*, *pparb*, *srebp1*, and *nr1h4*). Notably, 16S rRNA sequencing analysis revealed that *L. rhamnosus* differentially sculpted the gut microbiome composition and diversity in MCLR-exposed larvae, promoting the growth of beneficial genera like *Shewanella* and *Enterobacter* while inhibiting potential pathogens like *Vibrio*. Significant correlations were identified between specific gut bacterial taxa and host physiological parameters related to antioxidant capacity, lipid metabolism, and growth, suggesting that the modulation of gut microbiota by probiotics may contribute to their protective effects against MCLR toxicity. These findings demonstrate the potential of probiotics as an effective strategy for mitigating the adverse effects induced by MCLR.

## Data Availability

Data will be made available on request.

## Supplementary Materials

The following supporting information can be downloaded at https://www.mdpi.com/article/10.3390/toxics12070527/s1. Table S1: Sequences of primers for the tested genes in the present study.
