# Supplementary material for: Probiotics Alleviate Microcystin-LR-Induced Developmental Toxicity in Zebrafish Larvae
Source: Toxics. 2024 Jul 22;12(7):527. doi: 10.3390/toxics12070527 (PMC11280922; doi:10.3390/toxics12070527)
Supplement: Supplementary file 1 [file toxics-12-00527-s001.zip › toxics-3084007-supplementary.pdf]

Supporting Information

Probiotics alleviate microcystin-LR-induced developmental toxicity in zebrafish larvae

Qin Wu [a,b], Aoxue Gong [a], Xixia Liu [a,b], Jianjun Hou [a,b], Huan Liu [a,b], Ya Zhu [c*]

[a] Hubei Key Laboratory of Edible Wild Plants Conservation and Utilization, Huangshi Key Laboratory of Lake Biodiversity and Environmental Conservation, Hubei Normal University, Huangshi, Hubei province, 435002, China

[b] Hubei Engineering Research Center of Special Wild Vegetables Breeding and Comprehensive Utilization Technology, Huangshi, Hubei province, 435002, China

[c] School of Medicine, Taizhou University, Taizhou, Zhejiang province, 318000, China

***Author for correspondence:**

Email: zhuya@tzc.edu.cn (Y. Zhu)

Table S1.

Sequences of primers for the tested genes in the present study.

Gene name	Sequences of the primer (5'-3')	Accession number
gapdh	Forward: ctggtgacccgtgctgctt	NM_001115114
	Reverse: tttgccgccttctgcctta	
PPARa	Forward: catcttgccttgcagacatt	NM_001161333
	Reverse: cacgctcacttttcatttcac	
PPARb	Forward: gcgtaagctagtcgcaggtc	AF342937
	Reverse: tgcaccagagagtccatgtc	
srebp1	Forward: actcttctggtgtggctgct	NM_001105129
	Reverse: gagccttcagacacgtcctc	
nr1h4	Forward: cacaacaaacatcgcattcc	NM_001002574
	Reverse: gctgaagacttgggctgaac	
